# Rapid Progressing Allele HLA-B35 Px Restricted Anti-HIV-1 CD8+ T Cells Recognize Vestigial CTL Epitopes

**DOI:** 10.1371/journal.pone.0010249

**Published:** 2010-04-21

**Authors:** Christian B. Willberg, Keith E. Garrison, R. Brad Jones, Duncan J. Meiklejohn, Gerald Spotts, Teri J. Liegler, Mario A. Ostrowski, Annika C. Karlsson, Frederick M. Hecht, Douglas F. Nixon

**Affiliations:** 1 Division of Experimental Medicine, Department of Medicine, University of California San Francisco, San Francisco, California, United States of America; 2 Department of Immunology, University of Toronto, Toronto, Ontario, Canada; 3 Division of HIV/AIDS, Department of Medicine, University of California San Francisco, San Francisco, California, United States of America; 4 Department of Microbiology, Tumor and Cell Biology (MTC), Karolinska Institutet, Solna, Sweden; 5 Department of Virology, The Swedish Institute for Infectious Disease Control, Solna, Sweden; New York University, United States of America

## Abstract

**Background:**

The HLA-B*35-Px allele has been associated with rapid disease progression in HIV-1 infection, in contrast to the HLA-B*35-Py allele.

**Methodology/Principal Findings:**

Immune responses to two HLA-B*35 restricted HIV-1 specific CTL epitopes and their variants were followed longitudinally during early HIV-1 infection in 16 HLA-B*35+ individuals. Subjects expressing HLA-B*35-Px alleles showed no difference in response to the consensus epitopes compared to individuals with HLA-B*35-Py alleles. Surprisingly, all the HLA-B*35-Px+ individuals responded to epitope-variants even in the absence of a consensus response. Sequencing of the viral population revealed no evidence of variant virus in any of the individuals.

**Conclusions/Significance:**

This demonstrates a novel phenomenon that distinguishes individuals with the HLA-B*35-Px rapid progressing allele and those with the HLA-B*35-Py slower progressing allele.

## Introduction

Host genetics are known to have a major influence on HIV-1 disease progression [1], [2], [3], [4], [5]. For example, HLA-B*57 and -B*27 are two MHC class I alleles which have been associated with slower progression to AIDS [6], [7]. At the other end of the spectrum, HLA-B*35 alleles have been shown to be associated with more rapid disease progression [8].

HLA-*B35 alleles can be segregated into B*35-Px and -Py alleles, which differ by a number of amino acids located in the HLA binding groove [9]. These differences result in different peptide binding preferences. HLA-B*35-Py molecules preferentially bind peptides with a proline (P) at anchor residue 2 and a tyrosine (Y) at position 9. HLA-B*35-Px molecules also bind peptides with a proline at anchor residue 2, but can accept a much broader range of residues at position 9. However, both HLA-B*35-Px and -Py molecules are capable of binding epitopes that do not fit the conventional PY motif (Los Alamos Database, http://www.hiv.lanl.gov/). The most common B*35-Py alleles are B*3501 and B*3508, while the B*35-Px alleles include B*3502/3503/3504/5301.

The marked difference in HIV-1 disease progression of individuals expressing either HLA B*35-Py or -Px alleles is not understood. The overall magnitude of the HIV-1-specific CD8+ T cell immune response does not differ between individuals expressing either -Px or Py alleles [10]. However, this was measured at the level of the total CD8+ T cell response, and thus specific responses to individual epitopes were not differentiated. Measuring responses to specific epitopes may be critical in understanding the differences in disease progression between individuals with these alleles. We therefore set out to analyze T cell responses to two HLA-B*35 restricted HIV-1 epitopes in both HLA-B*35 -Px and -Py allele expressing individuals.

## Methods

This study was approved by the UCSF Institutional Review Board.

### Human Study Subjects and Blood Samples

Peripheral blood mononuclear cells (PBMC) samples were obtained from HIV-1 infected subjects, from the University of California San Francisco (UCSF) OPTIONS project (Table 1). HLA-A type, CD4 count and HIV-1 viral load were determined for each subject (Table 1). All samples were processed with Ficoll-Paque PLUS (Amersham Biosciences, Pittsburgh, PA) and PBMC were stored frozen in 10% DMSO in fetal bovine serum prior to subsequent analysis. Written informed consent and approval for this study was obtained in accordance with the guidelines of the Institutional Review Board.

**Table 1:** Clinical parameters, average CD4 and viral load (VL) were generated from across the entire study window.

B*35	Study ID	HLA-A		HLA-B		AverageCD4	AverageVL
	Subject 1	1	2407	3505	5107	401	52744
	Subject 2	2501	6801	702	3503	829	21644
**Px**	Subject 3	11	11	18	35	520	57035
	Subject 4	201	2402	3502	3924	492	15011
	Subject 5	2902	3101	3503	4403	826	3897
	Subject 7	1	2901	3518	8101	616	53262
	Subject 8	1	3	3528	51	728	59894
	Subject 9	1101	7401	3501	5101	1067	18713
	Subject 10	nt	nt	3501	4901		
**PY**	Subject 11	301	2601	3501	4001	597	72495
	Subject 12	201	2402	702	3501	993	45539
	Subject 13	201	1101	3501	4001	811	10324
	Subject 14	201	1101	3501	4402	645	4904
	Subject 15	201	1101	3501	5101	757	33454
	Subject 16	301	2402	3501	5701	430	19961
**PxPY**	Subject 6	1101	6801	3501	3505	705	20828

### ELISPOT assay

Quantification of HIV-specific T-cell responses using thawed viable PBMC was performed using the IFN-γ ELISPOT assay. Briefly, each well of a 96-well plate (Millipore MAHAS4510, Bedfort, MA) was coated with 50 µl of anti-IFN-γ mAb (Mabtech, Stockholm, Sweden) at 5 µg/ml. After incubation, each well was washed and blocked with 10% FCS in RPMI (Cellgro). PBMC (1×10^5^–2×10^5^) were added to duplicate wells and peptides were added at varying concentrations to the cells. As a positive control, the mitogen phytohemagglutinin (PHA) was used at 4 µg/ml, and wells with only media added were used as a negative control. After overnight incubation (14–16 hours) at 37°C, plates were washed with phosphate-buffered saline (PBS). Biotinylated anti-IFN-γ mAb 7-B6-1 (Mabtech) was added at 1 µg/ml, and incubated at 37°C for 1 hr. Plates were washed with PBS +0.1% Tween 20 and treated with streptavidin-bound alkaline phosphatase. After 1 h incubation, plates were washed with PBS +0.1% Tween 20 and developed using Alkaline Phosphatase Substrate Kit III (Vector Laboratories, Burlingame, CA). IFN-γ spot-forming units (SFUs) were visualized and counted using an AID EliSpot reader (Autoimmun Diagnostika GMBH, Germany). Spots were standardized to SFU/10^6^ PBMC. Spots formed in the presence of media alone were considered non-specific background and subtracted from the SFU in stimulated wells. The wells where the number of SFU was greater than two times background or greater than 50 SFU/106 PBMCs, which ever was higher, were considered as responses.

### Viral Load Assessment

Plasma HIV-1 viral load was determined by bDNA (Bayer).

## Results

T cell responses from 16 HLA-B*35+ HIV-1 infected individuals were analyzed using the IFN-γ enzyme-linked immunospot (ELISpot) assay over the first years of infection. All the patients were from the University of California San Francisco primary infection cohort (OPTIONS) [11]. Five individuals expressed an HLA-B*35-Px allele, and 10 individuals expressed an HLA-B*35-Py allele. One individual expressed both HLA B*35-Px and -Py alleles. No significant difference in CD4+ T cell count (p = 0.44) or plasma viral loads (p = 0.7) were observed between the two groups, as determined by two-tailed Mann Whitney test analysis (Table 1).

CD8+ T cell responses were determined by a standard IFN-γ ELISpot assay [12], [13], [14], using thawed peripheral blood mononuclear cells (PBMC). All spot numbers were normalized to numbers of IFN-γ spot-forming units (SFU) per 10^6^ PBMCs. Responses were determined by subtracting the SFU values from medium control wells from the peptide induced response. All experimental assays and analyses were conducted blinded to the individual's specific HLA-B*35 allele.

**Figure 1:**
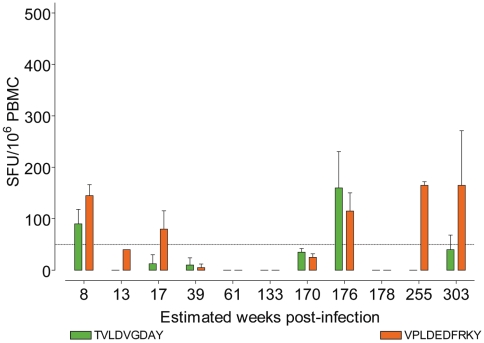
A representative example, from subject 1, of longitudinal CD8+ T cell IFN-γ responses to the TY9 epitope (solid green bars) and VY10 (solid orange bars). All subjects showed similar responses towards both epitopes that waxed and waned over the course of the study window.

PBMC were screened against two HLA-B*35 restricted HIV-1-Pol derived epitopes: TVLDVGDAY and VPLDEDFRKY [15], [16]. At one or more time points we observed a loss of an epitope specific-response in all individuals tested, an example is shown in Fig. 1. However, there was no clear difference in the size or number of epitope-specific responses between the either HLA-B*35-Px+ or -Py+ individuals (data not shown). HLA-B*35 -Px+ individuals did not display unusual CD4 counts or viral loads, a representative example shown from Subject 1 (E).

**Figure 2:**
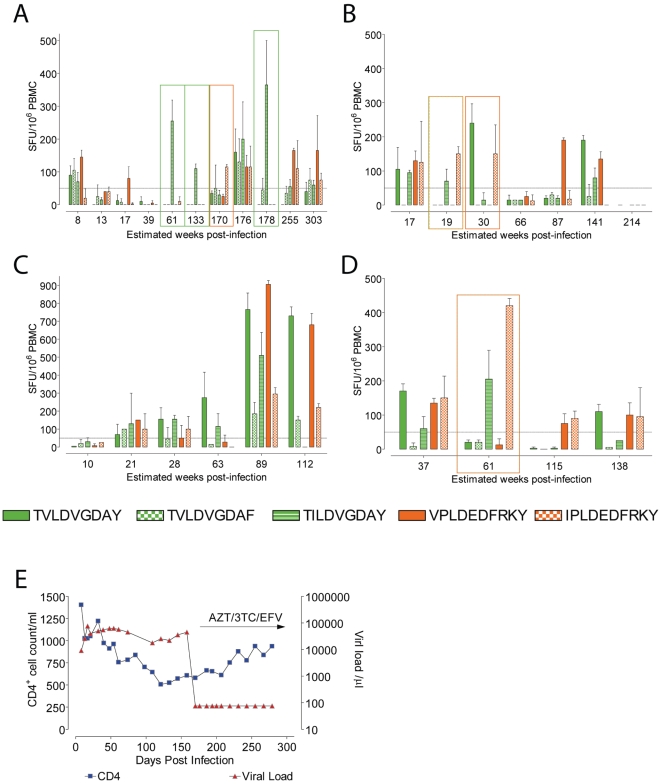
CD8+ T cell IFN-γ responses to both consensus epitopes (solid bars, TY9 green and VY10 orange), and variant epitopes (TY9 variants green, checkered or stripped bars, and VY10 variant orange checkered bars). Representative examples of longitudinal responses are shown for HLA-B*35 -Px+ Subjects 1 (A) and 2 (B), and HLA-B*35 -Py+ Subject 8 (C). CD8+ T cell IFN-γ responses from Subject 6, who expressed both HLA-B*35 -Px and -Py alleles (D). HLA-B*35 -Px+ individuals did not display unusual CD4 counts or viral loads, a representative example shown from Subject 1 (E).

Unexpectedly, all the HLA-B*35-Px+ individuals made responses to all the peptides tested. Strikingly, they were able to recognize the variant epitope in the absence of a consensus response at one or more time points tested, as shown for 2 patients (Subject 1 and Subject 2) (Fig. 2A and 2B). However, HLA-B*35-Py+ individuals never recognized the variant epitopes alone at any of the time points tested (Fig. 2C). Individuals who made responses solely to variant epitopes at one time point or more, showed no unexpected clinical characteristics at these or any other time points (Fig. 2E). We observed no difference in the magnitudes of responses targeting HLA-B*35 specific epitopes in either HLA-B*35 -Px+ or -Py+ individuals. Both groups were able to recognize and respond to epitope variants. However, only the HLA-B*35 -Px+ individuals were able to respond to variants in the absence of a wild-type response.

The ability of the five HLA-B*35 -Px+ individuals to make solely variant-specific responses implied that they may harbor variant HIV-1 within their viral population. To test this, their HIV-1 reverse transcriptase, codons 40 through 247, were sequenced using the TRUGENE HIV-1 RNA genotyping kit and OpenGene system software for sequence analysis (Siemens Medical Solutions Diagnostics) [17], [18]. At no time point tested, were any variants of the consensus epitopes seen (Table 2).

**Table 2:** HIV-1 Pol Sequencing of HLA-B*35 -Px allele expressing individuals.

Study ID	Estimate weeks Post Infection	T	V	L	D	V	G	D	A	Y	V	P	L	D	E	D	F	R	K	Y
Subject 1	653	.	.	.	.	.	.	.	.	.	.	.	.	.	K	.	.	.	.	.
Subject 2	92	.	.	.	.	.	.	.	.	.	.	.	.	.	K	X	.	.	.	.
Subject 3	182	.	.	.	.	.	.	.	.	.	.	.	.	.	.	.	.	.	.	.
Subject 4	10	.	.	.	.	.	.	.	.	.	.	.	.	.	.	N	.	.	.	.
	134	.	.	.	.	.	.	.	.	.	.	.	.	.	.	N	.	.	.	.
Subject 5	30	.	.	.	.	.	.	.	.	.	.	.	.	.	K	.	.	.	.	.
Subject 6	10	.	.	.	.	.	.	.	.	.	.	.	.	.	.	.	.	.	.	.
	13	.	.	.	.	.	.	.	.	.	.	.	.	.	.	.	.	.	.	.
	37	.	.	.	.	.	.	.	.	.	.	.	.	.	.	.	.	.	.	.
	61	.	.	.	.	.	.	.	.	.	.	.	.	.	X	X	.	.	.	.

Interestingly, one individual from the cohort, Subject 6, expressed both HLA-B*35 -Px and -Py alleles. This individual also responded solely to the variant epitopes at one time point (Fig. 2D). Again, this individual did not display variant viruses within their viral population. This suggests that the HLA-B*35-Px effect dominates over the HLA-B*35-Py influence. Lost or weak responses have been shown to be rescued by blocking PD-1 - PD-1L interactions. However, PD-1 - PD-1L blockage did not improve the restore absent responses in any of the individuals tested (data not shown).

## Discussion

HIV-1 responses associated with delayed disease progression have been shown to be efficiently cross-reactive with epitope variants [19]. We hypothesized that the individuals with HLA-B*35-Px alleles, associated with rapid disease progression, would have limited recognition of epitope variants. Thus, we predicted that HLA-B*35 -Py+ individuals would exhibit T-cell responses to variant peptides more frequently than individuals with HLA-B*35-Px alleles. Naturally occurring variants of both epitopes were tested: TVLDVGDAF, TILDVGDAY, and IPLDEDFRKY. HLA-B*35-Py+ individuals never recognized the variant epitopes alone at any of the time points tested. Unexpectedly, all the HLA-B*35-Px+ individuals made responses to all the peptides tested, and only the HLA-B*35 -Px+ individuals were able to respond to variants in the absence of a wild-type response, and no evidence for variant epitope viruses. We have thus named the responses to these epitopes “vestigial” CTL epitopes, as the responses are made to sequences that appear to be no longer present.

Due to the non-standard motifs of these epitopes, HLA tetramers could not be made and therefore individual CD8+ T cell populations could not be studied with this technology. This leaves open the question of whether the responses to the variant epitopes, made in the absence of a consensus epitope response, are unique responses specifically targeting the variant epitopes or cross-reactive responses that at these time points did not respond to the consensus epitope. Without variant viruses within the viral population to drive the variant-specific responses, it is intriguing to speculate on how specific responses could be generated and maintained. One possibility is that these variant epitopes are heteroclitic [20]; capable of stimulating CD8+ T cells to a greater magnitude than the consensus epitope against which they were originally generated. Since the two HLA-B*35 allele subtypes differ in their binding grooves, they may present epitopes in different orientations. Thus, at the time points where only responses to the variant (vestigial) epitopes are seen, the greater stimulus provided by the variant epitope could induce IFN-γ expression from otherwise unresponsive CD8+ T cells. This is an area that has been study in greater depth in the cancer immunity field [21], [22], [23].

This unusual phenomenon demonstrates a distinct difference between the HLA-B*35 alleles associated with rapid progression and those that are not. What role this plays in disease progression remains to be elucidated. Here, we provide the first evidence to our knowledge, of a difference in CD8+ T cell responses restricted to the two HLA-B*35 allele subtypes.

## References

[1] Kiepiela P, Leslie AJ, Honeyborne I, Ramduth D, Thobakgale C (2004). Dominant influence of HLA-B in mediating the potential co-evolution of HIV and HLA.. Nature.

[2] Fellay J, Shianna KV, Ge D, Colombo S, Ledergerber B (2007). A whole-genome association study of major determinants for host control of HIV-1.. Science.

[3] Dean M, Carrington M, Winkler C, Huttley GA, Smith MW (1996). Genetic restriction of HIV-1 infection and progression to AIDS by a deletion allele of the CKR5 structural gene. Hemophilia Growth and Development Study, Multicenter AIDS Cohort Study, Multicenter Hemophilia Cohort Study, San Francisco City Cohort, ALIVE Study.. Science.

[4] Carrington M, Bontrop RE (2002). Effects of MHC class I on HIV/SIV disease in primates.. AIDS.

[5] Ahuja SK, Kulkarni H, Catano G, Agan BK, Camargo JF (2008). CCL3L1-CCR5 genotype influences durability of immune recovery during antiretroviral therapy of HIV-1-infected individuals.. Nat Med.

[6] Carrington M, O'Brien SJ (2003). The influence of HLA genotype on AIDS.. Annu Rev Med.

[7] Scherer A, Frater J, Oxenius A, Agudelo J, Price DA (2004). Quantifiable cytotoxic T lymphocyte responses and HLA-related risk of progression to AIDS.. Proc Natl Acad Sci USA.

[8] Gao X, Nelson GW, Karacki P, Martin MP, Phair J (2001). Effect of a single amino acid change in MHC class I molecules on the rate of progression to AIDS.. N Engl J Med.

[9] Barber LD, Gillece-Castro B, Percival L, Li X, Clayberger C (1995). Overlap in the repertoires of peptides bound in vivo by a group of related class I HLA-B allotypes.. Curr Biol.

[10] Jin X, Gao X, Ramanathan M, Deschenes GR, Nelson GW (2002). Human immunodeficiency virus type 1 (HIV-1)-specific CD8+-T-cell responses for groups of HIV-1-infected individuals with different HLA-B*35 genotypes.. J Virol.

[11] Hecht FM, Busch MP, Rawal B, Webb M, Rosenberg E (2002). Use of laboratory tests and clinical symptoms for identification of primary HIV infection.. AIDS.

[12] Sandberg JK, Fast NM, Jordan KA, Furlan SN, Barbour JD (2003). HIV-specific CD8+ T cell function in children with vertically acquired HIV-1 infection is critically influenced by age and the state of the CD4+ T cell compartment.. J Immunol.

[13] Larsson M, Jin X, Ramratnam B, Ogg GS, Engelmayer J (1999). A recombinant vaccinia virus based ELISPOT assay detects high frequencies of Pol-specific CD8 T cells in HIV-1-positive individuals.. AIDS.

[14] Karlsson AC, Martin JN, Younger SR, Bredt BM, Epling L (2003). Comparison of the ELISPOT and cytokine flow cytometry assays for the enumeration of antigen-specific T cells.. Journal of Immunological Methods.

[15] Menéndez-Arias L, Mas A, Domingo E (1998). Cytotoxic T-lymphocyte responses to HIV-1 reverse transcriptase (review).. Viral Immunol.

[16] Sipsas NV, Kalams SA, Trocha A, He S, Blattner WA (1997). Identification of type-specific cytotoxic T lymphocyte responses to homologous viral proteins in laboratory workers accidentally infected with HIV-1.. J Clin Invest.

[17] Grant RM, Kuritzkes DR, Johnson VA, Mellors JW, Sullivan JL (2003). Accuracy of the TRUGENE HIV-1 genotyping kit.. J Clin Microbiol.

[18] Kuritzkes DR, Grant RM, Feorino P, Griswold M, Hoover M (2003). Performance characteristics of the TRUGENE HIV-1 Genotyping Kit and the Opengene DNA Sequencing System.. J Clin Microbiol.

[19] Turnbull EL, Lopes AR, Jones NA, Cornforth D, Newton P (2006). HIV-1 epitope-specific CD8+ T cell responses strongly associated with delayed disease progression cross-recognize epitope variants efficiently.. J Immunol.

[20] Nicholson LB, Anderson AC, Kuchroo VK (2000). Tuning T cell activation threshold and effector function with cross-reactive peptide ligands.. International Immunology.

[21] Gold JS, Ferrone CR, Guevara-Patiño JA, Hawkins WG, Dyall R (2003). A single heteroclitic epitope determines cancer immunity after xenogeneic DNA immunization against a tumor differentiation antigen.. J Immunol.

[22] Dyall R, Bowne WB, Weber LW, LeMaoult J, Szabo P (1998). Heteroclitic immunization induces tumor immunity.. The Journal of Experimental Medicine.

[23] Brown ME, Miao H, McKee MD (2007). Recognition of carcinoembryonic antigen peptide and heteroclitic peptide by peripheral blood T lymphocytes.. J Immunother.

