# Correction: Rapid Progressing Allele HLA-B35 Px Restricted Anti-HIV-1 CD8+ T Cells Recognize Vestigial CTL Epitopes

**DOI:** 10.1371/journal.pone.0160293

**Published:** 2016-07-25

**Authors:** Christian B. Willberg, Keith E. Garrison, R. Brad Jones, Duncan A. Meiklejohn, Gerald Spotts, Teri J. Liegler, Mario A. Ostrowski, Annika C. Karlsson, Frederick M. Hecht, Douglas F. Nixon

The fourth author’s name is incorrect. The correct name is: Duncan A. Meiklejohn. The correct citation is: Willberg CB, Garrison KE, Jones RB, Meiklejohn DA, Spotts G, Liegler TJ, et al. (2010) Rapid Progressing Allele HLA-B35 Px Restricted Anti-HIV-1 CD8+ T Cells Recognize Vestigial CTL Epitopes. PLoS ONE 5(4): e10249. doi:10.1371/journal.pone.0010249
